# Percentile curves for cardiometabolic disease markers in Canadian children and youth: a cross-sectional study

**DOI:** 10.1186/s12887-018-1289-2

**Published:** 2018-09-28

**Authors:** Nicole Ata, Bryan Maguire, David C Hamilton, Stefan Kuhle

**Affiliations:** 1 Departments of Pediatrics and Obstetrics & Gynaecology, Dalhousie University, Halifax, NS Canada; 2 Department of Mathematics and Statistics, Dalhousie University, Halifax, NS Canada

**Keywords:** Child, Adolescent, Metabolism, Obesity, Development, Diabetes

## Abstract

**Background:**

The objective of this study to develop percentile curves for cardiometabolic disease markers in a population-based sample of Canadian children and youth.

**Methods:**

The analysis used data from 6116 children and adolescents between 6 and 19 years of age who participated in the Canadian Health Measures Survey cycles 1 (2007/2009), 2 (2009/2011), and 3 (2012/2013). Total cholesterol, HDL cholesterol, and hemoglobin A1c levels as well as fasting levels of triglycerides, insulin, and homeostasis model assessment insulin resistance were measured using standardized procedures. Age- and sex-specific centiles for all markers were calculated using Cole and Green’s LMS method.

**Results:**

With the exception of hemoglobin A1c, all markers showed age- and sex-related differences during childhood and adolescence.

**Conclusions:**

We have developed centile curves for cardiometabolic disease markers in Canadian children and adolescents and demonstrated age and sex differences that should be considered when evaluating these markers in this age group.

## Background

Cardiovascular disease (CVD) is currently the leading cause of death worldwide [1]. With the exception of congenital heart disease, CVD manifests in adulthood, but its risk factors are already detectable in childhood. Abnormal blood lipids and diabetes are among the risk factors for the development of CVD [2, 3]. An abnormal lipid profile can include elevated total cholesterol, elevated triglycerides, and low high-density lipoprotein (HDL) cholesterol. Insulin resistance plays an important role in the development of youth-onset type 2 diabetes, an emerging disease in children and youth [4]. Homeostasis model assessment estimates insulin resistance (HOMA-IR) from fasting levels of insulin and glucose [5]; other measures that have been used to identify insulin resistance or diabetes include fasting insulin and glycosylated hemoglobin (HbA1c), respectively [6–8].

Levels of these markers vary by sex and across age in childhood and adolescence, and percentile curves have been developed to describe their physiologic development. Percentile curves have been published for lipids and markers of insulin resistance in various populations [9–14]. Since these curves are specific to populations and there are no percentile curves for the levels of these markers in Canadian children, the objective of this study was to develop percentile curves for cardiometabolic markers in a population-based sample of Canadian children and youth.

## Methods

### Study design

This study used data of children and youth aged 6 to 19 years from the Canadian Health Measures Survey (CHMS) cycles 1 to 3, a representative, cross-sectional survey assessing health and wellness in Canadians [15–17]. The survey includes a household interview to obtain sociodemographic and health information and a visit to a mobile examination centre to perform physical measurements and tests. The sampling frame of the Canadian Labour Force Survey was used to identify the collection sites for the mobile examination centres. Within each collection site, households were selected using the 2006 Census as the sampling frame. Interviews and examinations for the CHMS Cycle 1 were performed between 2007 and 2009, for Cycle 2 between 2009 and 2011, and for Cycle 3 between 2012 and 2013. Household response rates were 69.6, 75.9, and 74.1%, respectively; final response rates in the 3 cycles were 51.7, 55.7 and 51.7%, respectively [15–17]. We combined data from the 3 cycles as per Statistics Canada guidelines [18]. A total of 11,999 persons participated in physical examination part of the three survey cycles. The present analysis uses data from 6116 children and adolescents between 6 and 19 years of age.

The Health Canada Research Ethics Board gave approval for the CHMS. All participants gave written informed consent; parents or guardians consented on behalf of children aged 6 to 13 years, and the child provided their assent to participate; youth 14 to 17 years consented on their own, but their parents or guardians had to give verbal permission for the household interview [15]. The current project was approved by the IWK Health Centre Research Ethics Board, Halifax, NS, Canada (File # 1014413).

### Laboratory measurements

Blood for measurement of cardiometabolic markers was collected by standard venipuncture. Fasted blood samples for measurement of insulin, glucose, and triglycerides were taken in a randomly selected sample of participants. The sample was obtained by randomly offering each respondent a clinic appointment either in the morning (after an overnight fast) or in the afternoon (non-fasted) [15]. Blood samples were centrifuged within 2 h and aliquoted within 4 h of collection. The samples were stored either in the refrigerator or in the freezer until shipping. Samples were shipped once a week to the Health Canada reference laboratory in Ottawa. Participants with diabetes were excluded from the analysis of insulin, HOMA-IR, and HbA1c; participants taking lipid-lowering medication were excluded from the analysis of lipids. Levels of total cholesterol, HDL cholesterol, triglycerides, and glucose were measured using a colorimetric test and HbA1c was measured using a immunoturbidimetric test on the Vitros 5,1FS (Ortho Clinical Diagnostics, Markham, ON, Canada). Fasting insulin levels were determined using a solid-phase, two-site chemiluminescent immunometric assay on the Advia Centaur XP (Siemens, Erlangen, Germany). Since insulin measurements in cycle 1 were performed using a different method and had a considerable proportion of levels below the test’s limit of detection, we only used insulin measurements from cycles 2 and 3 in the present analysis. Fasting insulin and glucose levels were used to calculate HOMA-IR as (fasting insulin [μU/L] x glucose [mmol/L]) / 22.5 [19].

### Statistical analysis

Percentile curves for total cholesterol, HDL cholesterol, triglycerides, insulin, HOMA-IR, and HbA1c were modeled using the LMS method by Cole and Green [20]. We have described the LMS method in detail elsewhere [21]. Briefly, the method uses a Box-Cox transformation to normalize the data and models the mean (M), variance (S), and skewness (L) as parameters over age using cubic splines. Centiles and z-scores for the truncated standard normal distribution can then be determined from the three parameters at each age [20]. We calculated the 3rd, 10th, 25th, 50th, 75th, 90th, and 97th centile for each marker. Models were fit to data from respondents up to age 30 years to avoid unusual behaviour of the spline functions near the end of the age range. The goodness of fit for each model was assessed using residual quantile plots (“worm plots”) [22]. All calculations were performed using sample weights provided by Statistics Canada to account for the design effect and reduce non-response bias. The statistical software package R [23] with the *gamlss* package [24] was used to perform the statistical analyses.

## Results

**Fig. 1:**
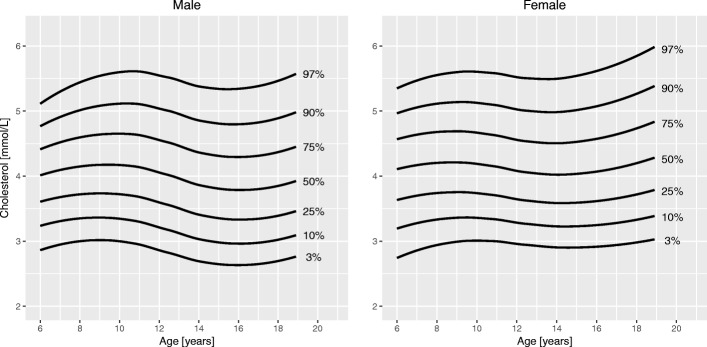
Percentile curves for total cholesterol levels for male and female Canadian children and youth aged 6 to 19 years

**Fig. 2:**
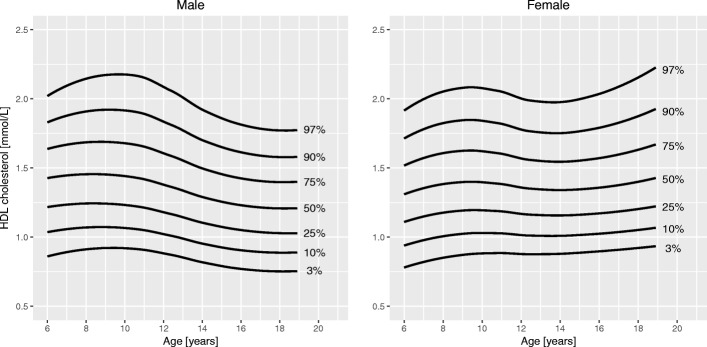
Percentile curves for high-density lipoprotein (HDL) cholesterol levels for male and female Canadian children and youth aged 6 to 19 years

**Fig. 3:**
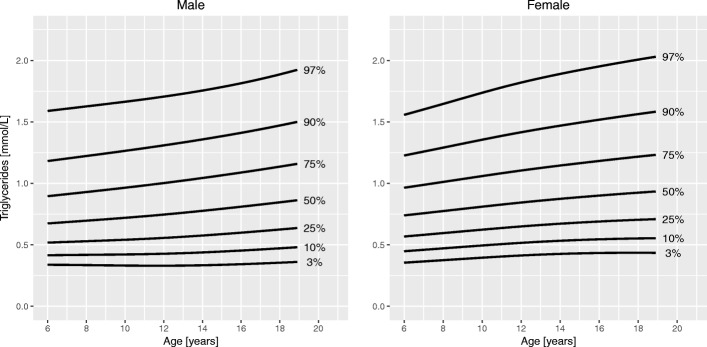
Percentile curves for triglyceride levels for male and female Canadian children and youth aged 6 to 19 years

**Fig. 4:**
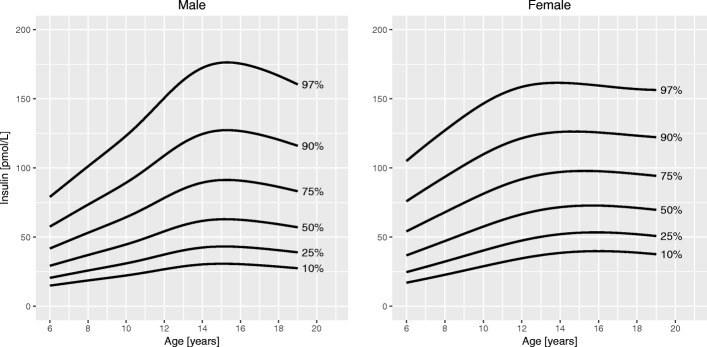
Percentile curves for insulin levels for male and female Canadian children and youth aged 6 to 19 years

**Fig. 5:**
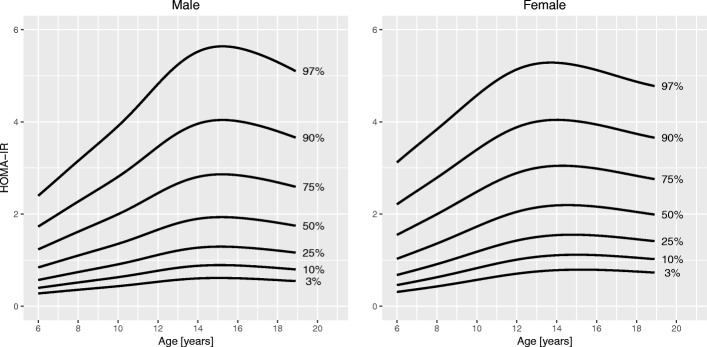
Percentile curves for homeostasis model assessment insulin resistance (HOMA-IR) levels for male and female Canadian children and youth aged 6 to 19 years

**Fig. 6:**
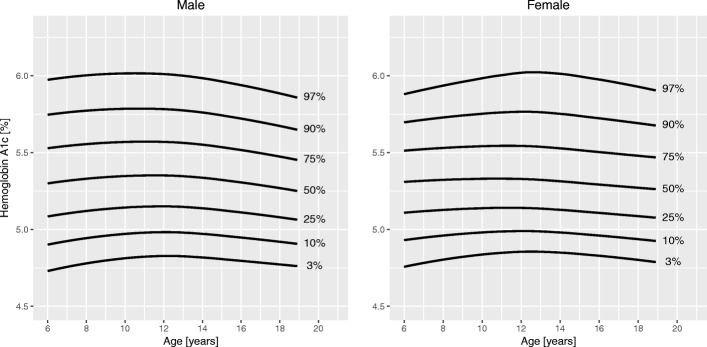
Percentile curves for hemoglobin A1c levels for male and female Canadian children and youth aged 6 to 19 years

Sociodemographic characteristics of the sample are summarized in Table 1. Figures 1, 2, 3, 4, 5 and 6 and Tables 2, 3, 4, 5, 6 and 7 show the percentile curves and their values for total cholesterol, HDL cholesterol, triglycerides, insulin, HOMA-IR, and HbA1c.

**Table 1:** Characteristics of 6116 Canadian children and youth aged 6 to 19 years in the Canadian Health Measures Survey Cycles 1 to 3

	Prevalence [%]
Sex	
Male	51.6
Female	48.4
Region of Canada	
Atlantic Canada	6.7
Québec	22.5
Ontario	40.5
Prairies	18.2
British Columbia	12.2
Racial origin	
White	80.9
Black	5.9^a^
Asian	10.7^a^
Other	2.4^a^
Weight status (IOTF)	
Underweight	7.5
Normal weight	65.6
Overweight	17.4
Obese	9.5
Household education	
Secondary school or less	15.7
College	46.6
University	37.6
Household income	
$30,000 or less	15.1
$30,001 - $60,000	22.8
$60,001 - $80,000	18.3
$80,001 - $100,000	16.3
> $100,000	27.5

*Abbreviations*: *IOTF* International Obesity Task Force

^a^Coefficient of variation between 16.6 and 33.3%; interpret with caution as per Statistics Canada sampling variability reporting guidelines

**Table 2:** L, M, and S values, and percentiles of total cholesterol [mmol/L] by age and sex for Canadian children and youth aged 6 to 19 years

Sex	Age	n	L	M	S	3rd	10th	25th	50th	75th	90th	97th
Female	6	203	1.2662	4.0824	0.1676	2.73	3.18	3.61	4.08	4.54	4.94	5.32
	7	184	1.0339	4.1657	0.1666	2.85	3.27	3.70	4.17	4.63	5.05	5.46
	8	215	0.7986	4.2255	0.1654	2.96	3.35	3.76	4.23	4.70	5.14	5.58
	9	233	0.5790	4.2369	0.1645	3.01	3.38	3.78	4.24	4.72	5.17	5.63
	10	256	0.3985	4.2026	0.1642	3.02	3.37	3.75	4.20	4.68	5.14	5.62
	11	283	0.2725	4.1427	0.1646	3.00	3.33	3.70	4.14	4.62	5.09	5.58
	12	187	0.2000	4.0742	0.1658	2.95	3.28	3.64	4.07	4.55	5.02	5.51
	13	178	0.1564	4.0321	0.1673	2.92	3.24	3.60	4.03	4.51	4.98	5.48
	14	170	0.1308	4.0222	0.1691	2.91	3.23	3.59	4.02	4.50	4.98	5.49
	15	175	0.1173	4.0342	0.1717	2.90	3.23	3.59	4.03	4.53	5.01	5.54
	16	178	0.1121	4.0701	0.1746	2.91	3.24	3.61	4.07	4.58	5.08	5.62
	17	186	0.1066	4.1326	0.1769	2.94	3.28	3.66	4.13	4.65	5.17	5.73
	18	168	0.1017	4.2050	0.1788	2.99	3.33	3.72	4.21	4.74	5.27	5.85
	19	152	0.0901	4.2612	0.1809	3.02	3.37	3.77	4.26	4.81	5.36	5.96
Male	6	203	1.1968	4.0369	0.1508	2.86	3.24	3.62	4.04	4.44	4.80	5.15
	7	211	0.8365	4.0884	0.1523	2.95	3.30	3.67	4.09	4.51	4.90	5.29
	8	232	0.5410	4.1350	0.1551	3.01	3.35	3.71	4.13	4.58	4.99	5.42
	9	239	0.3792	4.1691	0.1597	3.03	3.37	3.73	4.17	4.63	5.08	5.54
	10	263	0.3624	4.1766	0.1651	3.00	3.35	3.73	4.18	4.66	5.12	5.61
	11	277	0.3556	4.1469	0.1703	2.95	3.30	3.69	4.15	4.64	5.12	5.62
	12	207	0.3332	4.0711	0.1757	2.87	3.22	3.61	4.07	4.57	5.06	5.57
	13	207	0.3217	3.9598	0.1801	2.77	3.12	3.50	3.96	4.46	4.95	5.46
	14	205	0.2648	3.8546	0.1833	2.69	3.02	3.40	3.85	4.35	4.84	5.36
	15	184	0.1878	3.7910	0.1863	2.64	2.97	3.34	3.79	4.29	4.79	5.32
	16	208	0.1426	3.7735	0.1886	2.62	2.95	3.32	3.77	4.28	4.79	5.33
	17	183	0.1015	3.7992	0.1883	2.65	2.98	3.34	3.80	4.31	4.82	5.38
	18	144	0.0557	3.8565	0.1873	2.70	3.03	3.40	3.86	4.37	4.90	5.47
	19	139	0.0083	3.9299	0.1869	2.76	3.09	3.46	3.93	4.46	4.99	5.58

*Abbreviations*: *L* lambda (skewness); *M* mu (mean); *S* sigma (variance)

**Table 3:** L, M, and S values, and percentiles of HDL cholesterol [mmol/L] by age and sex for Canadian children and youth aged 6 to 19 years

Sex	Age	n	L	M	S	3rd	10th	25th	50th	75th	90th	97th
Female	6	203	0.6653	1.2995	0.2301	0.78	0.94	1.10	1.30	1.51	1.70	1.90
	7	184	0.5810	1.3513	0.2315	0.82	0.98	1.15	1.35	1.57	1.78	1.99
	8	215	0.4894	1.3925	0.2317	0.85	1.01	1.18	1.39	1.62	1.84	2.07
	9	233	0.4025	1.4128	0.2304	0.88	1.03	1.20	1.41	1.64	1.87	2.11
	10	256	0.3317	1.4069	0.2269	0.89	1.04	1.20	1.41	1.63	1.86	2.10
	11	283	0.2946	1.3835	0.2222	0.89	1.03	1.19	1.38	1.60	1.82	2.05
	12	187	0.2932	1.3527	0.2180	0.87	1.01	1.16	1.35	1.56	1.77	1.99
	13	178	0.2666	1.3372	0.2152	0.87	1.00	1.15	1.34	1.54	1.74	1.96
	14	170	0.1991	1.3404	0.2145	0.88	1.01	1.16	1.34	1.55	1.75	1.98
	15	175	0.1202	1.3502	0.2156	0.89	1.02	1.17	1.35	1.56	1.77	2.01
	16	178	0.0477	1.3590	0.2180	0.90	1.03	1.17	1.36	1.57	1.79	2.04
	17	186	−0.0261	1.3764	0.2213	0.91	1.04	1.19	1.38	1.60	1.83	2.09
	18	168	−0.0758	1.4019	0.2258	0.92	1.05	1.20	1.40	1.63	1.88	2.16
	19	152	−0.0551	1.4209	0.2316	0.92	1.06	1.22	1.42	1.66	1.92	2.21
Male	6	203	0.8228	1.4346	0.2226	0.86	1.04	1.22	1.43	1.65	1.85	2.06
	7	211	0.6385	1.4440	0.2207	0.89	1.06	1.23	1.44	1.66	1.87	2.09
	8	232	0.4626	1.4509	0.2215	0.91	1.07	1.24	1.45	1.68	1.89	2.12
	9	239	0.3069	1.4533	0.2256	0.92	1.07	1.24	1.45	1.69	1.92	2.17
	10	263	0.1937	1.4465	0.2296	0.92	1.07	1.24	1.45	1.68	1.93	2.19
	11	277	0.1465	1.4230	0.2297	0.91	1.05	1.22	1.42	1.66	1.90	2.16
	12	207	0.1687	1.3827	0.2281	0.89	1.02	1.18	1.38	1.61	1.84	2.09
	13	207	0.2352	1.3335	0.2266	0.85	0.99	1.14	1.33	1.55	1.77	2.00
	14	205	0.3157	1.2882	0.2251	0.82	0.95	1.10	1.29	1.49	1.70	1.92
	15	184	0.3915	1.2523	0.2244	0.79	0.92	1.07	1.25	1.45	1.64	1.85
	16	208	0.4549	1.2270	0.2256	0.77	0.90	1.05	1.23	1.42	1.61	1.81
	17	183	0.4843	1.2122	0.2260	0.75	0.89	1.03	1.21	1.40	1.59	1.78
	18	144	0.4698	1.2062	0.2245	0.75	0.89	1.03	1.21	1.40	1.58	1.77
	19	139	0.4299	1.2084	0.2234	0.76	0.89	1.03	1.21	1.40	1.58	1.78

*Abbreviations*: *L* lambda (skewness); *M* mu (mean); *S* sigma (variance)

**Table 4:** L, M, and S values, and percentiles of triglycerides [mmol/L] by age and sex for Canadian children and youth aged 6 to 19 years

Sex	Age	n	L	M	S	3rd	10th	25th	50th	75th	90th	97th
Female	6	78	−0.0198	0.7390	0.3934	0.35	0.45	0.57	0.74	0.96	1.23	1.56
	7	85	− 0.0360	0.7567	0.3932	0.36	0.46	0.58	0.76	0.99	1.26	1.60
	8	98	−0.0522	0.7744	0.3931	0.37	0.47	0.60	0.77	1.01	1.29	1.65
	9	127	−0.0680	0.7922	0.3931	0.39	0.48	0.61	0.79	1.04	1.32	1.69
	10	114	−0.0821	0.8100	0.3931	0.40	0.49	0.62	0.81	1.06	1.35	1.74
	11	139	−0.0928	0.8273	0.3932	0.40	0.51	0.64	0.83	1.08	1.39	1.78
	12	93	−0.0987	0.8438	0.3935	0.41	0.52	0.65	0.84	1.10	1.42	1.82
	13	88	− 0.0996	0.8593	0.3942	0.42	0.52	0.66	0.86	1.13	1.44	1.86
	14	88	−0.0958	0.8738	0.3954	0.43	0.53	0.67	0.87	1.14	1.47	1.89
	15	91	−0.0874	0.8874	0.3972	0.43	0.54	0.68	0.89	1.16	1.49	1.92
	16	91	−0.0746	0.9003	0.3996	0.43	0.54	0.69	0.90	1.18	1.52	1.95
	17	89	−0.0581	0.9125	0.4026	0.43	0.55	0.70	0.91	1.20	1.54	1.98
	18	74	−0.0389	0.9240	0.4061	0.44	0.55	0.70	0.92	1.22	1.56	2.01
	19	72	−0.0180	0.9346	0.4102	0.43	0.55	0.71	0.93	1.23	1.58	2.03
Male	6	102	−0.2816	0.6737	0.4053	0.34	0.41	0.52	0.67	0.90	1.18	1.59
	7	98	−0.2348	0.6849	0.4111	0.34	0.42	0.52	0.68	0.91	1.20	1.61
	8	116	−0.1881	0.6961	0.4169	0.33	0.42	0.53	0.70	0.93	1.22	1.63
	9	120	−0.1418	0.7075	0.4227	0.33	0.42	0.54	0.71	0.95	1.24	1.64
	10	133	−0.0967	0.7195	0.4281	0.33	0.42	0.54	0.72	0.96	1.26	1.66
	11	135	−0.0544	0.7322	0.4329	0.33	0.42	0.55	0.73	0.98	1.29	1.68
	12	96	−0.0165	0.7459	0.4368	0.33	0.43	0.56	0.75	1.00	1.31	1.71
	13	112	0.0157	0.7606	0.4395	0.33	0.43	0.57	0.76	1.02	1.33	1.73
	14	97	0.0421	0.7763	0.4412	0.33	0.44	0.58	0.78	1.04	1.36	1.75
	15	93	0.0630	0.7927	0.4422	0.34	0.45	0.59	0.79	1.07	1.38	1.78
	16	103	0.0788	0.8098	0.4428	0.34	0.45	0.60	0.81	1.09	1.41	1.81
	17	92	0.0899	0.8275	0.4431	0.35	0.46	0.61	0.83	1.11	1.44	1.85
	18	78	0.0968	0.8457	0.4435	0.35	0.47	0.62	0.85	1.14	1.47	1.89
	19	74	0.0999	0.8642	0.4441	0.36	0.48	0.64	0.86	1.16	1.50	1.93

*Abbreviations*: *L* lambda (skewness); *M* mu (mean); *S* sigma (variance)

**Table 5:** L, M, and S values, and percentiles of insulin [pmol/L] by age and sex for Canadian children and youth aged 6 to 19 years

Sex	Age	n	L	M	S	3rd	10th	25th	50th	75th	90th	97th
Female	6	74	0.0810	36.8032	0.5822	11.69	17.05	24.69	36.80	54.17	75.94	105.08
	7	85	0.0832	41.9738	0.5661	13.77	19.86	28.47	41.97	61.13	84.90	116.43
	8	95	0.0861	47.1805	0.5505	15.95	22.79	32.35	47.18	68.00	93.58	127.19
	9	124	0.0909	52.4480	0.5352	18.25	25.83	36.33	52.45	74.82	102.02	137.42
	10	110	0.0997	57.6269	0.5201	20.59	28.91	40.32	57.63	81.35	109.87	146.55
	11	136	0.1121	62.4301	0.5044	22.90	31.91	44.13	62.43	87.17	116.52	153.78
	12	92	0.1263	66.5218	0.4884	25.06	34.66	47.52	66.52	91.86	121.50	158.64
	13	88	0.1414	69.6293	0.4734	26.89	36.93	50.22	69.63	95.15	124.61	161.05
	14	86	0.1553	71.6632	0.4607	28.26	38.58	52.12	71.66	97.07	126.08	161.58
	15	86	0.1652	72.6769	0.4515	29.11	39.56	53.17	72.68	97.82	126.30	160.90
	16	90	0.1703	72.8110	0.4465	29.42	39.88	53.45	72.81	97.67	125.70	159.62
	17	86	0.1707	72.2282	0.4460	29.21	39.58	53.04	72.23	96.85	124.61	158.18
	18	74	0.1665	71.1378	0.4500	28.57	38.80	52.10	71.14	95.65	123.38	157.03
	19	71	0.1592	69.7465	0.4583	27.60	37.65	50.80	69.75	94.31	122.28	156.42
Male	6	98	−0.0050	29.2964	0.5269	10.90	14.93	20.54	29.30	41.81	57.62	79.12
	7	95	−0.0012	33.2034	0.5309	12.24	16.82	23.21	33.20	47.50	65.58	90.17
	8	116	0.0028	37.0587	0.5348	13.54	18.66	25.83	37.06	53.14	73.49	101.18
	9	118	0.0065	40.8164	0.5385	14.78	20.44	28.37	40.82	58.67	81.26	112.00
	10	130	0.0097	44.7223	0.5419	16.06	22.28	31.01	44.72	64.42	89.36	123.31
	11	129	0.0126	49.2160	0.5451	17.54	24.40	34.05	49.22	71.02	98.66	136.29
	12	95	0.0153	54.1827	0.5477	19.18	26.75	37.41	54.18	78.32	108.91	150.58
	13	110	0.0178	58.6671	0.5499	20.65	28.87	40.44	58.67	84.91	118.19	163.49
	14	95	0.0203	61.7110	0.5519	21.61	30.27	42.47	61.71	89.42	124.55	172.38
	15	91	0.0232	62.9131	0.5539	21.91	30.75	43.23	62.91	91.27	127.21	176.13
	16	102	0.0271	62.5506	0.5561	21.65	30.46	42.90	62.55	90.85	126.71	175.45
	17	91	0.0318	61.1922	0.5583	21.03	29.67	41.90	61.19	88.97	124.15	171.90
	18	76	0.0370	59.3041	0.5602	20.25	28.65	40.54	59.30	86.30	120.44	166.70
	19	74	0.0431	57.1325	0.5615	19.39	27.51	39.00	57.13	83.18	116.05	160.46

*Abbreviations*: *L* lambda (skewness); *M* mu (mean); *S* sigma (variance)

**Table 6:** L, M, and S values, and percentiles of HOMA-IR by age and sex for Canadian children and youth aged 6 to 19 years

Sex	Age	n	L	M	S	3rd	10th	25th	50th	75th	90th	97th
Female	6	74	0.0680	1.0304	0.6120	0.31	0.46	0.68	1.03	1.55	2.21	3.12
	7	85	0.0816	1.1930	0.5955	0.37	0.54	0.79	1.19	1.77	2.50	3.48
	8	95	0.0953	1.3607	0.5795	0.43	0.63	0.91	1.36	2.00	2.79	3.84
	9	124	0.1090	1.5413	0.5642	0.50	0.73	1.04	1.54	2.24	3.09	4.21
	10	110	0.1226	1.7284	0.5496	0.57	0.83	1.18	1.73	2.48	3.40	4.57
	11	136	0.1354	1.9044	0.5360	0.64	0.93	1.31	1.90	2.71	3.67	4.90
	12	92	0.1467	2.0481	0.5235	0.71	1.01	1.43	2.05	2.89	3.88	5.14
	13	88	0.1557	2.1435	0.5125	0.75	1.07	1.50	2.14	3.00	4.01	5.26
	14	86	0.1619	2.1886	0.5036	0.78	1.11	1.54	2.19	3.05	4.04	5.28
	15	86	0.1645	2.1908	0.4970	0.79	1.12	1.55	2.19	3.04	4.02	5.23
	16	90	0.1635	2.1612	0.4930	0.79	1.11	1.54	2.16	2.99	3.94	5.12
	17	86	0.1591	2.1092	0.4916	0.78	1.09	1.50	2.11	2.91	3.84	5.00
	18	74	0.1514	2.0465	0.4928	0.75	1.05	1.46	2.05	2.83	3.74	4.87
	19	71	0.1411	1.9837	0.4965	0.73	1.02	1.41	1.98	2.75	3.65	4.77
Male	6	98	0.0558	0.8427	0.5725	0.28	0.40	0.57	0.84	1.23	1.73	2.40
	7	95	0.0576	0.9718	0.5752	0.32	0.46	0.66	0.97	1.43	2.00	2.78
	8	116	0.0595	1.1004	0.5778	0.36	0.52	0.74	1.10	1.62	2.27	3.15
	9	118	0.0610	1.2255	0.5802	0.40	0.57	0.82	1.23	1.80	2.54	3.52
	10	130	0.0617	1.3545	0.5823	0.44	0.63	0.91	1.35	2.00	2.81	3.91
	11	129	0.0619	1.5014	0.5841	0.48	0.70	1.01	1.50	2.22	3.12	4.35
	12	95	0.0618	1.6614	0.5855	0.53	0.77	1.11	1.66	2.45	3.46	4.82
	13	110	0.0618	1.8031	0.5864	0.58	0.84	1.21	1.80	2.67	3.76	5.24
	14	95	0.0619	1.8974	0.5873	0.60	0.88	1.27	1.90	2.81	3.96	5.52
	15	91	0.0627	1.9332	0.5883	0.61	0.89	1.29	1.93	2.86	4.04	5.63
	16	102	0.0646	1.9204	0.5896	0.61	0.89	1.28	1.92	2.84	4.02	5.60
	17	91	0.0673	1.8754	0.5910	0.59	0.86	1.25	1.88	2.78	3.93	5.48
	18	76	0.0705	1.8126	0.5922	0.57	0.83	1.21	1.81	2.69	3.80	5.30
	19	74	0.0747	1.7406	0.5929	0.54	0.80	1.16	1.74	2.58	3.64	5.08

Abbreviations: *L* lambda (skewness); *M* mu (mean); *S* sigma (variance)

**Table 7:** L, M, and S values, and percentiles of hemoglobin A1c by age and sex for Canadian children and youth aged 6 to 19 years

Sex	Age	n	L	M	S	3rd	10th	25th	50th	75th	90th	97th
Female	6	192	0.6293	0.0531	0.0565	0.0476	0.0493	0.0511	0.0531	0.0552	0.0570	0.0589
	7	178	0.0341	0.0532	0.0563	0.0478	0.0495	0.0512	0.0532	0.0552	0.0571	0.0591
	8	204	−0.5398	0.0532	0.0561	0.0480	0.0496	0.0513	0.0532	0.0553	0.0573	0.0593
	9	224	−1.0745	0.0533	0.0560	0.0482	0.0497	0.0513	0.0533	0.0553	0.0574	0.0596
	10	248	−1.5959	0.0533	0.0560	0.0484	0.0498	0.0514	0.0533	0.0554	0.0575	0.0598
	11	275	−2.1144	0.0533	0.0559	0.0485	0.0499	0.0514	0.0533	0.0554	0.0576	0.0600
	12	184	−2.5527	0.0533	0.0558	0.0486	0.0499	0.0514	0.0533	0.0554	0.0577	0.0602
	13	172	−2.8025	0.0532	0.0557	0.0486	0.0499	0.0514	0.0532	0.0554	0.0576	0.0603
	14	166	−2.8388	0.0531	0.0555	0.0485	0.0498	0.0513	0.0531	0.0553	0.0575	0.0601
	15	167	−2.7545	0.0530	0.0553	0.0484	0.0497	0.0512	0.0530	0.0551	0.0574	0.0599
	16	172	−2.6052	0.0529	0.0551	0.0483	0.0496	0.0511	0.0529	0.0550	0.0572	0.0597
	17	177	−2.4044	0.0528	0.0550	0.0481	0.0495	0.0509	0.0528	0.0549	0.0570	0.0595
	18	165	−2.1906	0.0527	0.0550	0.0480	0.0494	0.0509	0.0527	0.0548	0.0569	0.0593
	19	149	−2.0342	0.0527	0.0552	0.0479	0.0493	0.0508	0.0527	0.0548	0.0569	0.0592
Male	6	195	−0.4642	0.0530	0.0619	0.0473	0.0490	0.0509	0.0530	0.0553	0.0575	0.0598
	7	205	−0.5917	0.0532	0.0612	0.0476	0.0492	0.0510	0.0532	0.0554	0.0576	0.0599
	8	227	−0.7384	0.0533	0.0605	0.0478	0.0494	0.0512	0.0533	0.0555	0.0577	0.0600
	9	229	−0.8769	0.0534	0.0598	0.0480	0.0496	0.0513	0.0534	0.0556	0.0578	0.0601
	10	256	−0.9903	0.0535	0.0591	0.0481	0.0497	0.0514	0.0535	0.0557	0.0578	0.0601
	11	267	−1.0740	0.0535	0.0584	0.0482	0.0498	0.0515	0.0535	0.0557	0.0579	0.0602
	12	204	−1.1009	0.0535	0.0580	0.0483	0.0498	0.0515	0.0535	0.0557	0.0578	0.0601
	13	201	−1.0887	0.0535	0.0577	0.0483	0.0498	0.0515	0.0535	0.0556	0.0578	0.0600
	14	203	−1.0467	0.0534	0.0574	0.0482	0.0497	0.0514	0.0534	0.0555	0.0576	0.0599
	15	180	−1.0081	0.0532	0.0571	0.0481	0.0496	0.0512	0.0532	0.0554	0.0574	0.0596
	16	205	−0.9894	0.0530	0.0567	0.0479	0.0494	0.0511	0.0530	0.0551	0.0572	0.0594
	17	180	−1.0085	0.0528	0.0561	0.0478	0.0493	0.0509	0.0528	0.0549	0.0569	0.0591
	18	138	−1.0586	0.0527	0.0555	0.0477	0.0492	0.0508	0.0527	0.0547	0.0567	0.0588
	19	135	−1.1174	0.0526	0.0549	0.0477	0.0491	0.0507	0.0526	0.0546	0.0566	0.0587

*Abbreviations*: *L* lambda (skewness); *M* mu (mean); *S* sigma (variance)

**Total cholesterol** curves had a bimodal distribution for both boys and girls. Overall, cholesterol levels were slightly higher in girls than in boys. Median levels at 6 years were 4.1 mmol/L in girls and 4.0 mmol/L in boys. In boys, the 50th centile peaked at age 10 years (4.2 mmol/L). The lowest median cholesterol level in boys was seen at 16 years of age (3.8 mmol/L), after which it increased to 3.9 mmol/L at 19 years. In girls, the 50th centile for cholesterol had a peak at 9 years (4.2 mmol/L), decreased to a trough at 15 years (4.0 mmol/L) and increased again to 4.3 mmol/L at 19 years of age. **HDL cholesterol** showed a bimodal distribution in girls, but only one peak in boys. In boys, the median levels were highest before 11 years (1.5 mmol/L) and then steadily declined until 19 years (1.2 mmol/L). Median HDL cholesterol in girls peaked at age 10 years (1.4 mmol/L) and after a trough (1.3 mmol/L) increased again to 1.4 mmol/L at 19 years. Median levels of **triglycerides** exhibited a steady linear increase from 6 years (0.7 mmol/L) to 19 years (0.9 mmol/L) for both sexes. **Insulin** levels were overall higher in girls than in boys. For both sexes, median levels increased until about 14 years of age (62 and 72 pmol/L in boys and girls, respectively), after which they slightly decreased to 57 pmol/L in boys and 70 pmol/L in girls at 19 years of age. Centile curves for **HOMA-IR** largely mirrored those for insulin with the 50th percentile peaking at 15 years for both sexes (1.9 for boys and 2.2 for girls). Median **HbA1c** levels held nearly constant around 5.3% from 6 to 19 years for both sexes.

## Discussion

The objective of this study was to develop percentile curves for total cholesterol, HDL cholesterol, triglycerides, insulin, HOMA-IR, and HbA1c in a population-based sample of Canadian children and youth. We found age- and sex-related differences in blood levels for all markers except for HbA1c.

A bimodal shape of the centile curves for total cholesterol levels has been described in various Western populations [9, 11, 13, 25]. A pre-adolescent peak at around 8 to 10 years of age that is more pronounced in boys is followed by a decrease during adolescence and another peak in late adolescence and young adulthood. The same pattern, but without a post-pubertal rise in boys, can be seen for HDL cholesterol [9, 11, 13]. The pubertal trough of cholesterol levels may be the result of the well described insulin resistance during puberty [26]. Clinicians should be aware of these physiologic changes when interpreting cholesterol levels. However, it should also be acknowledged that median levels of cholesterol in our study as well as in other studies varied by 10% or less in either direction during childhood and adolescence [9, 11, 13, 25].

Median triglyceride levels in our sample showed a nearly linear increase by about 30% from around 0.7 to 0.9 mmol/L in both sexes during childhood and adolescence. Some investigators previously described a bimodal pattern in girls with peaks at around 12 and 19 years of age [9, 11], while others also reported the linear increase we found [13]. These differences may be explained by different degrees of smoothing applied during the modeling process.

Median fasting insulin levels were higher in girls than in boys, and levels in both sexes peaked at around 15 years of age followed by a slight decrease towards late adolescence. Similar age and sex differences have also been reported by others [13, 27]. The peak in puberty reflects the physiologically reduced insulin sensitivity and concomitant increase in insulin secretion during that period [28, 29]. The median insulin levels in our study and others [12, 13, 27] varied with pubertal levels ranging from 52 to 63 pmol/L in boys and from 65 to 73 pmol/L in girls. These differences may be explained by differences in the insulin assay used [30] or differences in the body composition, ethnicity, and puberty stage of the children in the sample. The shape of the HOMA-IR curves was similar to those for fasting insulin. Schwartz et al. found a significant correlation between fasting insulin and HOMA-IR but found both only modestly correlated with the insulin resistance measurement gold standard, the euglycemic-hyperinsulinemic glucose clamp [31]. HOMA-IR still is among the most commonly used surrogate measure of insulin resistance to date. Given the variation over sex and age, in particular the physiologic insulin resistance in puberty, the use of an age- and sex-specific percentile-based cutoff for HOMA-IR is warranted. Unfortunately, such a cutoff has not been established to date [32].

Glycosylated hemoglobin or HbA1c is an established marker for long-term glycemic control in patients with diabetes [33]. HbA1c has been proposed as a screening tool for undiagnosed diabetes in adults [6] and children with overweight or obesity [8], but the evidence is still very limited. We found very little change in HbA1c levels from childhood to late adolescence, and there was no difference between the sexes. To the best of our knowledge, only three previous studies have examined HbA1c levels during childhood [12, 14, 34]. Only Peplies et al. in the European IDEFICS cohort developed percentile curves to describe the changes in levels across age and found a 15% increase in median HbA1c levels between 7 and 11 years for both sexes [12].

The strengths of our study include the use of a large population-based sample and the use of standardized protocols and procedures for the measurement of the cardiometabolic marker levels. A shortcoming of the use of cross-sectional data is that it is not clear if the trajectories of individual children follow this pattern; longitudinal data may be more accurate in describing age-related changes but are considerably more resource intensive to collect at the population level. Due to the relatively small proportion of visible minority children in the sample (< 20%), we were not able to investigate ethnic differences in marker levels and trajectories. Another limitation of our study is that we were unable to take puberty stage, which may influence insulin and lipid levels, into account as this information was not available in the CHMS. By contrast to some of the previous studies in this area, we did not restrict our analysis to children with a healthy weight [9, 12], as our goal was to describe population-based trajectories. Since the inclusion of overweight and obese children in our sample may have influenced lipid and insulin levels, our percentiles cannot be considered as reference values.

## Conclusions

Our study has developed percentile curves for cardiometabolic disease markers in Canadian children and adolescents. We have demonstrated age- and sex-related differences in marker levels for lipids, insulin, and HOMA-IR that should be considered when evaluating these markers in children and adolescents.

## Abbreviations

CHMS: Canadian Health Measures Survey
CVD: Cardiovascular disease
HbA1c: Hemoglobin A1c
HDL: High-density lipoprotein
HOMA-IR: Homeostasis model assessment insulin resistance
LMS: Lambda Mu Sigma
